# Serum miRNA levels are related to glucose homeostasis and islet autoantibodies in children with high risk for type 1 diabetes

**DOI:** 10.1371/journal.pone.0191067

**Published:** 2018-01-18

**Authors:** Linda Åkerman, Rosaura Casas, Johnny Ludvigsson, Beatriz Tavira, Camilla Skoglund

**Affiliations:** 1 Division of Pediatrics, Department of Clinical and Experimental Medicine, Faculty of Medicine and Health Sciences, Linköping University, Linköping, Sweden; 2 Crown Princess Victoria Children´s Hospital, University Hospital, Linköping, Region Östergötland, Sweden; 3 Division of Drug Research, Department of Medical and Health Sciences, Faculty of Medicine and Health Sciences, Linköping University, Linköping, Sweden; University of Massachusetts Medical School, UNITED STATES

## Abstract

Micro RNAs (miRNAs) are promising disease biomarkers due to their high stability. Their expression in serum is altered in type 1 diabetes, but whether deviations exist in individuals with high risk for type 1 diabetes remains unexplored. We therefore assessed serum miRNAs in high-risk individuals (n = 21) positive for multiple islet autoantibodies, age-matched healthy children (n = 17) and recent-onset type 1 diabetes patients (n = 8), using Serum/Plasma Focus microRNA PCR Panels from Exiqon. The miRNA levels in the high-risk group were similar to healthy controls, and no specific miRNA profile was identified for the high-risk group. However, serum miRNAs appeared to reflect glycemic status and ongoing islet autoimmunity in high-risk individuals, since several miRNAs were associated to glucose homeostasis and autoantibody titers. High-risk individuals progressing to clinical disease after the sampling could not be clearly distinguished from non-progressors, while miRNA expression in the type 1 diabetes group deviated significantly from high-risk individuals and healthy controls, perhaps explained by major metabolic disturbances around the time of diagnosis.

## Introduction

Micro RNA (miRNA) molecules provide an additional layer of regulation in protein synthesis and affect a wide range of physiological and pathological processes such as development, differentiation, autoimmunity and cancer [1–3]. Over 2500 miRNAs have been described in humans [4], creating a complex regulatory network since each single miRNA can bind to several different mRNAs. These molecules are expressed to a different degree in different tissues [5], and while the majority are found within cells, they can also be detected in serum [6]. Micro RNA molecules are very stable in cell-free body fluids like serum, with high resistance to RNAase digestion and with an ability to remain intact in extreme conditions like extended storage and repeated freeze-thaw [6]. This makes them very appealing as minimally invasive potential biomarkers of disease [7].

Considering their omnipresence, it is likely that miRNAs are involved in the regulation of genes related to development also of type 1 diabetes (T1D). There are indeed reports on differentially expressed miRNAs in peripheral blood mononuclear cells (PBMC) [8–11] and in specific immune cell subsets like regulatory T cells [12] from T1D patients. Alterations in serum levels have also been observed in newly diagnosed T1D, where some specific miRNAs appear to be related to glycemic control [13, 14]. Experimental data has shown altered levels of miRNAs within the islets of non-obese diabetic (NOD) mice during the pre-diabetic phase, and the expression of specific miRNAs was affected when exposing both murine- and human islets to pro-inflammatory cytokines [15]. Other than a recent report on altered miRNA expression in CD4+ T cell subsets of first-degree relatives of T1D patients with positivity for multiple autoantibodies [16], data on miRNA expression during the pre-diabetic period is scarce. We therefore aimed to study whether deviations of miRNA levels in serum could be detected in children positive for multiple islet autoantibodies, considered at high risk of T1D, recruited from the general Swedish population.

## Materials and methods

### Subjects

Characteristics for the subjects included in the study are given in Table 1. Children with high risk of T1D (n = 21) were identified by islet autoantibodies screening among samples from 17055 participants in the ABIS (All Babies in Southeast Sweden) cohort, described in detail elsewhere [17]. The definition of high risk was positivity for 2 or 3 different islet autoantibodies on at least two of the sampling occasions at age 1, 2.5–3, 5–6 and 8. GADA and IA2A were tested at all time points and IAA at age 5–6 and 8. The high-risk individuals participated in a two-year prospective follow-up study involving blood sampling every 6 months for measurement of fasting blood glucose, autoantibodies, C-peptide, HbA1c and for HLA-genotyping, and an oral glucose tolerance test (OGTT) once every year [18]. The serum samples used for miRNA quantification in this study were from the baseline visit of the follow-up, which for the high-risk individuals who later developed T1D meant before disease onset.

**Table 1:** Individual characteristics of T1D high-risk subjects, T1D patients and healthy controls included in the study.

							Autoantibody status *						
Gender	Age at sampling	Fasting C-peptide	HbA1c	f- glucose	OGTT	Weight	GADA	IA2A	IAA		ZnT8A		HLA-risk
	*(years)*	*(nmol/l)*	*(%)*	*(mmol/l)*	*(mmol/l)*	*(kg)*				Tryp	Arg	Glut	
**High-risk**													
Male	11.5	0.34	4.2	5.6	9.1	33	**32860**	BC	**13.2**	BC	BC	BC	Moderate
Female	11.7	0.32	4.3	5.3	10.6	45.2	**262.5**	BC	**5.3**	BC	BC	BC	Low
Male	10.5	0.27	4.6	4.9	6.9	39.6	BC	**444.5**	**11.9**	**416.8**	**396.7**	**215.7**	Low
Male	10.6	0.43	3.6	7	6.9	39.5	**179.8**	**740**	**8**	**153.7**	**142.6**	**72.3**	Low
Male	10.2	0.33	4	5.3	6	35.4	**200.8**	**6930**	BC	**2832**	BC	BC	Moderate
Male	11.6	0.32	4.5	5.4	8.1	58.7	**161**	**1128**	**11.9**	**324**	**1967**	**347.6**	Moderate
Male	10.9	0.39	4.5	6	6.4	37.2	**6400**	BC	**4.4**	BC	**270.6**	BC	Moderate
Female	10.0	0.91	4.2	5.2	6.1	50.8	**8730**	BC	**17.6**	**787.8**	**220.3**	**68.6**	Moderate
Male	10.0	0.32	4.5	5.2	8.3	32.6	**140.4**	**329.5**	BC	BC	**468**	BC	Moderate
Male	9.9	0.39	4.3	5	6.2	39.2	**3782**	BC	BC	BC	**1620**	BC	Moderate
Female	10.9	0.34	3.8	5.2	7.2	35.8	**768**	**346**	BC	**45.3**	**23400**	**38.6**	Moderate
Male	10.4	0.20	4.2	5.1	5.8	44.4	**94.7**	**272.5**	**6.7**	**33.6**	**1237**	**45.8**	Low
Male	10.3	0.44	4.4	5.1	5.8	56	**4540**	BC	**6.7**	BC	BC	BC	High
Male	11.4	0.29	4.5	6.6	7.4	30	**545**	BC	**82.4**	BC	BC	BC	Low
Male	11.2	0.36	4.7	5.2	9.1	39.6	BC	**256.5**	**7**	BC	BC	BC	Moderate
Male	11.2	0.47	4	5.5	6.1	42.4	**589**	**1530**	**56.8**	BC	**2245**	BC	High
Female	11.4	0.33	4.2	5.2	9.9	48.3	**1742**	**325.5**	**15.4**	BC	**214**	BC	Low
Female	9.6	0.25	4.4	4.3	6.2	26.7	**80.9**	**18.9**	BC	**34**	**242.5**	BC	High
Female	10.5	0.31	4.5	5.9	11.1	34.5	**8770**	**12.7**	BC	BC	BC	BC	High
Female	10.6	0.46	4.5	5.7	6.2	43.7	**8120**	BC	**6.2**	BC	BC	BC	Low
**Type 1 diabetes**													
Female	13.7	0.04	7.8	N/A	N/A	48.2	BC	**167.1**	BC	**33.2**	**127.6**	**23.6**	Moderate
Female	10.9	0.30	9.5	N/A	N/A	49.0	BC	**1332**	**5.2**	**76.1**	**77.0**	**59.2**	Moderate
Female	9.9	0.17	9	N/A	N/A	39.9	BC	**108.9**	BC	**43.2**	**54.6**	**29.2**	Moderate
Female	12.1	0.17	9.1	N/A	N/A	30.4	**8100**	**787**	**13.4**	**2011**	**49.7**	**30.4**	High
Female	11.2	0.26	8.1	N/A	N/A	46.2	**1427**	**834**	BC	**1026.5**	**1307**	**965**	Moderate
Female	12.4	0.07	7.2	N/A	N/A	34.4	**1410**	**44.5**	BC	BC	BC	BC	Low
Male	11.8	0.07	6.3	N/A	N/A	38.5	**68.3**	BC	**27.5**	**59.0**	**184.1**	**55.6**	High
Male	11.2	0.43	7.5	N/A	N/A	40.5	BC	**35.1**	BC	**27.6**	BC	BC	Low
**Healthy controls**													
Male	11.9	0.60	N/A	N/U	N/A	30.5	BC	BC	BC	BC	BC	BC	Moderate
Male	12.0	0.34	N/A	N/U	N/A	39.8	BC	BC	BC	BC	BC	BC	Low
Male	12.0	0.44	N/A	N/U	N/A	35	BC	BC	BC	BC	BC	BC	Low
Male	12.0	0.35	N/A	N/U	N/A	40.9	BC	BC	BC	BC	BC	BC	Low
Male	12.4	0.35	N/A	N/U	N/A	43.3	BC	BC	BC	BC	BC	BC	High
Male	12.4	0.69	N/A	N/U	N/A	35	BC	BC	BC	BC	BC	BC	Low
Male	12.1	0.32	N/A	N/U	N/A	42.8	BC	BC	BC	BC	BC	BC	Low
Male	12.2	0.39	N/A	N/U	N/A	43	BC	BC	BC	BC	BC	BC	Low
Female	10.8	0.54	N/A	N/U	N/A	41	BC	BC	BC	BC	BC	BC	Low
Female	10.7	0.51	N/A	N/U	N/A	51	BC	BC	BC	BC	BC	BC	Low
Male	12.3	0.55	N/A	N/U	N/A	54.6	BC	BC	BC	BC	BC	BC	Low
Female	11.5	0.57	N/A	N/U	N/A	35.2	BC	BC	BC	BC	BC	BC	Low
Female	11.5	0.41	N/A	N/U	N/A	50.7	BC	BC	BC	BC	BC	BC	Low
Female	11.6	0.89	N/A	N/U	N/A	45.3	BC	BC	BC	BC	BC	BC	Low
Male	11.7	0.47	N/A	N/U	N/A	36.3	BC	BC	BC	BC	BC	BC	Low
Female	12.3	0.56	N/A	N/U	N/A	50.8	BC	BC	BC	BC	BC	BC	Low
Male	12.1	0.36	N/A	N/U	N/A	48.3	BC	BC	N/A	N/A	N/A	N/A	N/A

OGTT refers to the 2 hour value. N/A = not available. N/U = not used. BC = below cut-off

* The serum levels used as cut-offs for autoantibody positivity for GADA, IA2, IAA and ZnT8 (tryptophane/arginine/glutamine) were 63.8; 5.8; 4.1; 32; 23.1 and 26 units/ml, respectively. Samples exceeding these cut-offs are indicated with boldfaced font and samples below cut-off are presented as BC. Serum samples obtained 0 or 4 days after disease onset were used for autoantibody analysis in diabetic children, to avoid measurement of IAA induced by insulin treatment.

Healthy controls (n = 17) were selected among participants in ABIS. The criteria to be regarded as healthy were as follows: negative for islet autoantibodies, no diabetes type 1 or 2, or any autoimmune disorder, no asthma, eczema or allergies, and no first-degree relatives with diabetes or autoimmune disorders. Samples from recent-onset T1D patients (1 month after onset, n = 8) were obtained from the pediatric clinic at Linköping University Hospital, Sweden.

### Sample preparation

Fasting serum samples were collected in Z Serum Clot Activator tubes (Vacuette, Greiner Bio-One), during the morning hours to avoid diurnal effects. Due to different locations of the participants, some samples could not be processed immediately after sampling. To avoid that this would interfere with our results, all samples were left at room temperature over night before processing. Samples from the high-risk- and healthy children were kept at -70°C, while samples from T1D patients were stored at -20°C.

### Assessment of relative miRNA levels

RNA was extracted from serum using miRCURY™ RNA Isolation Kit for Biofluids (Exiqon), according to manufacturer’s protocol, using MS2 bacteriophage RNA (Roche) as a carrier and including a protein precipitation- and DNAse digestion step. Before extraction, sera was thawed at room temperature and centrifuged for 5 min at 3000xg, and only the uppermost part was used. A fixed volume of serum was used for each sample (200 μl) and cDNA was produced from the entire volume of eluted RNA, using Universal cDNA Synthesis Kit (Exiqon) according to instructions provided by the manufacturer. RNA quality was assessed by PCR-based QC panels (miRCURY ™ microRNA QC PCR Panel, Exiqon) before proceeding to the miRNA profiling. One sample (from the high-risk group) was excluded after the QC-panel due to haemolysis.

Relative miRNA quantities were obtained by 384-well miRCURY LNA™ Universal RT microRNA PCR Serum/Plasma Focus Panels (Exiqon, S1 Table). Each serum sample was analysed in duplicate in two separate plates, using cDNA from two different RT-reactions. Applied Biosystem´s 7900HT Fast Real-Time PCR System was used for sequence detection, and threshold cycle (Ct) values were obtained by sequence detection systems (SDS) version 2.3 (Applied Biosystems). Cycling conditions were set as specified by the manufacturer, including a dissociation step. Automatic baseline adjustment was applied, and a common threshold was set for all reactions in the study. To establish individual detection cut-off values for each miRNA, a cDNA-sample from a mock-RNA purification on a clean water sample was run in duplicate for all primer sets. Only genes for which >60% of the samples had detectable levels were included in further data processing. Dissociation curves were inspected to exclude non-specific reactions or wells with primer-dimer formation. Twenty-two miRNAs were excluded from the analyses because the results from the mock-RNA panels indicated unspecific amplification, and another 28 miRNAs were excluded because the levels were undetectable in more than 60% of the samples. The remaining miRNAs (n = 129) were included in data pre-processing and statistical analyses.

### Statistics

GenEx Professional, version 5.4.2 (MultiD Analyses AB) was used for pre-processing of raw data. Raw Ct values were normalized to the global mean, and the normalized expression of each miRNA was converted to relative quantities on the linear scale by assigning the normalized Ct-values as N, according to: N = 2^(Ctrel-Ct)^, where Ctrel was 0. Log2 was then applied. Data were mean centred for principal component analysis. Statistical analysis of pre-processed data was performed in GenEx and IBM SPSS Statistics 22 for Windows, and graphs were prepared with GraphPad Prism 5 for Windows. Since parts of the data set deviated from Gaussian distribution, as assessed by the Shapiro-Wilk test, non-parametric tests were used. Mann-Whitney U-test was used for group comparisons, and correlation between miRNA expression and clinical/laboratory variables was assessed by Spearman's rank correlation coefficient. This study was a hypothesis generating study and therefore correction of p-values for multiple comparisons were not performed and thus differences were considered statistically significant at p <0.05. There were no significant differences in gender or weight between T1D, high-risk individuals and healthy controls. The age in the high-risk group was significantly lower compared to T1D and healthy controls (p = 0.033 and p<0.001, respectively, median age high-risk 10.6, T1D 11.5 and healthy 12.0 years). However, correlation analysis revealed that age correlated only with a few miRNAs, and these had no effect on the results.

### miRNA pathway analysis

miRWalk v2.0 (http://zmf.umm.uni-heidelberg.de/apps/zmf/mirwalk2/) [19] was used to predict significant miRNA interactions with genes associated to pathways, ontologies and disorders via the Encyclopedia of Genes and Genomes (KEGG) and Gene Ontologies (GO) [20]. In short, the analysis involves identifying genes targeted by specific miRNAs, followed by target gene annotation based on involvement in signaling pathways and biological processes, and subsequent statistical testing for over-representation of target gene sets within a certain biological process or pathway. In the KEGG pathway analysis, FDR-correction was used to adjust for multiple testing, with the corrected p-value cut-off set to <0.05. Pathways were included only when the target genes were significantly over-represented according to both KEGG- and GO databases.

### Ethical considerations

All participating children and responsible guardians were thoroughly informed and provided a written informed consent before participation. The Research Ethics Committee of the Faculty of Health Sciences at Linköping University in Sweden has given ethical approval for the ABIS study (Dnr 36287 and Dnr 03–092), for the study of high risk individuals within ABIS (Dnr M13-09 and Dnr M52-05) and for studying samples from T1D patients (Dnr 02–483).

## Results

### Serum miRNAs in high-risk individuals and reference groups

**Fig 1:**
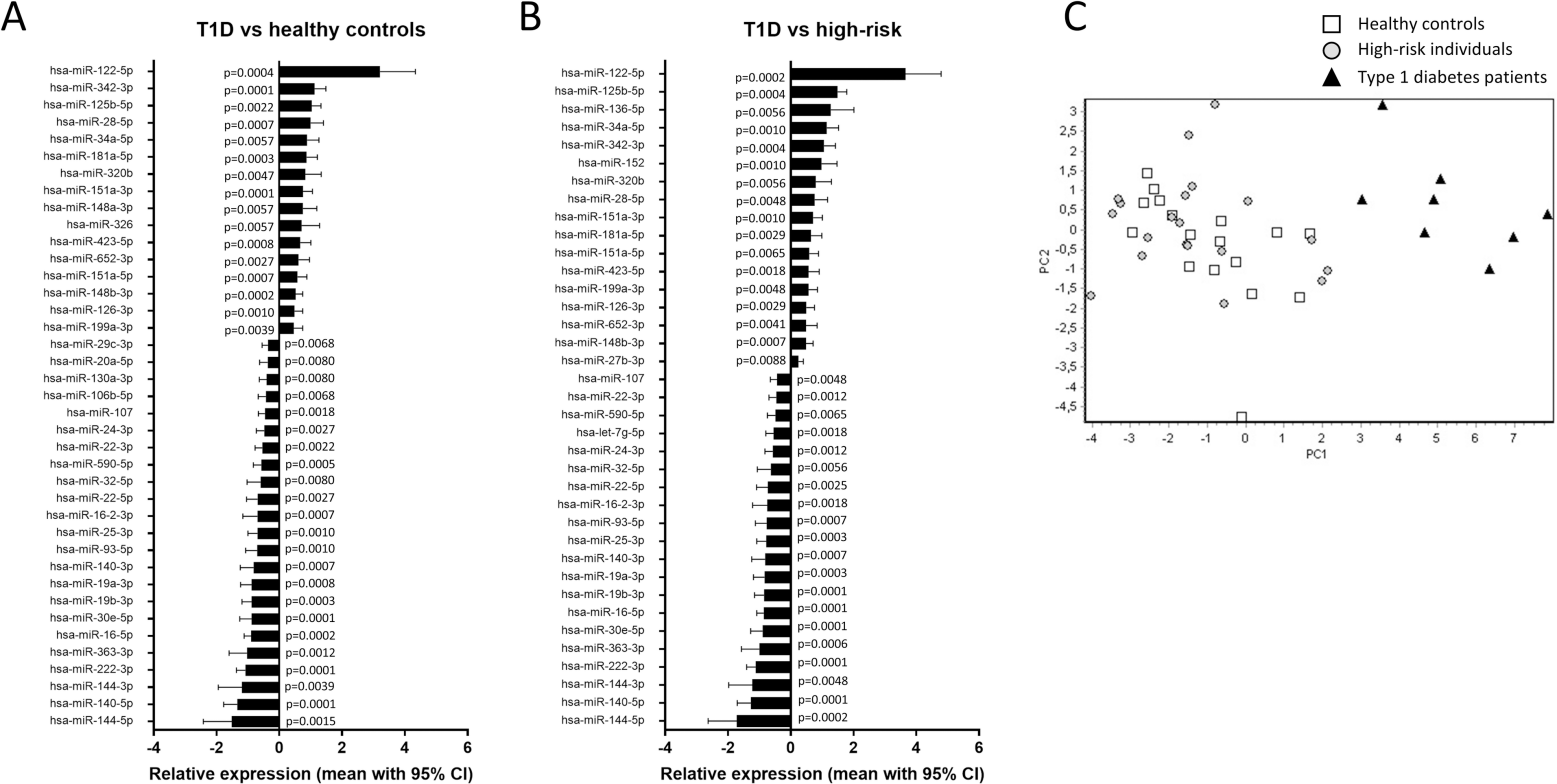
Relative expression and principal component analysis (PCA) of miRNAs in serum. Relative expression in **A)** children recently diagnosed with T1D (1 month post-onset, n = 8) relative to individuals with high risk of T1D (n = 20, set to 0 in the graph) and **B)** T1D in relation to healthy controls (n = 17, set to 0 in the graph). In the figure, miRNAs with a p-value of <0.01 are presented. **C)** PCA visualization (score plot) based on the 58 miRNAs differing between children with T1D (black triangles) and healthy controls (empty squares), also including T1D high-risk individuals (grey circles).

Serum levels of 129 miRNAs were similar in high-risk individuals (n = 20) and healthy controls (n = 17) (S1 File). Only two miRNAs differed between the groups: miR-497-5p was downregulated in high-risk individuals (p = 0.034) and miR-339-3p was upregulated (p = 0.043). The individuals with high HLA risk within the high-risk group (n = 4) had increased levels of miR-339-3p (p = 0.009) and the low HLA risk individuals (n = 7) had decreased levels of miR-497-5p (p = 0.005) compared to healthy controls. Comparison of high-risk- and recent onset T1D children (n = 8) revealed that 60 miRNAs differed significantly between these groups (Fig 1A, S1 File). The comparison between T1D- and healthy subjects showed that 58 miRNAs differed between them (Fig 1B, S1 File). Reflecting the similarity between high-risk individuals and healthy controls, we observed an overlap of 80% in the miRNAs that were dysregulated in T1D in relation to healthy- and high-risk children. A PCA score plot including the miRNAs differing significantly between T1D and healthy controls showed that the diabetic group was clearly separated from the healthy controls (Fig 1C). When including high-risk individuals in the same plot, they were evenly scattered among the healthy children. In the high-risk group, two miRNAs differed between high HLA risk individuals (n = 4) and low HLA risk individuals (n = 7): miR-148a-3p was upregulated in high HLA risk (p = 0.042) and miR-93-3p was downregulated in high HLA risk (p = 0.024).

### miRNAs and progression/non-progression to clinical disease among high-risk individuals

**Fig 2:**
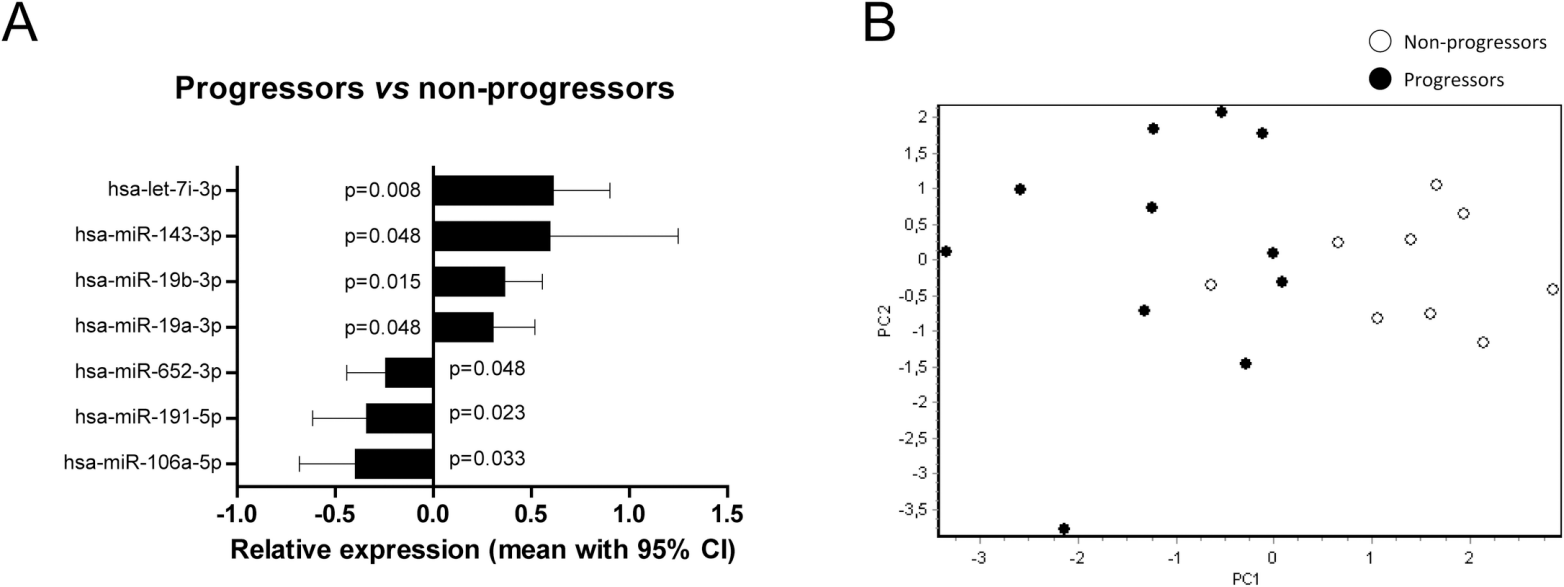
Relative expression and PCA visualization of miRNAs in serum from high-risk children that developed diabetes (progressors) relative to those who did not (non-progressors). **A)** Expression in progressors (n = 11) relative to non-progressors (n = 9) whose mean expression is set to 0 in the graph. **B)** PCA score plot based on the seven miRNAs that differed between progressors (black circles) and non-progressors (empty circles).

We next assessed whether miRNA profile could distinguish high-risk individuals who later were diagnosed with T1D (progressors, n = 11) from those who remained symptom-free (non-progressors, n = 9, August 2016). Seven of the 129 miRNAs differed between progressors and non-progressors (p<0.05, Fig 2A), and inclusion of them in a PCA score plot showed separation between the groups (Fig 2B).

### miRNAs and dysglycaemia among high-risk individuals

**Fig 3:**
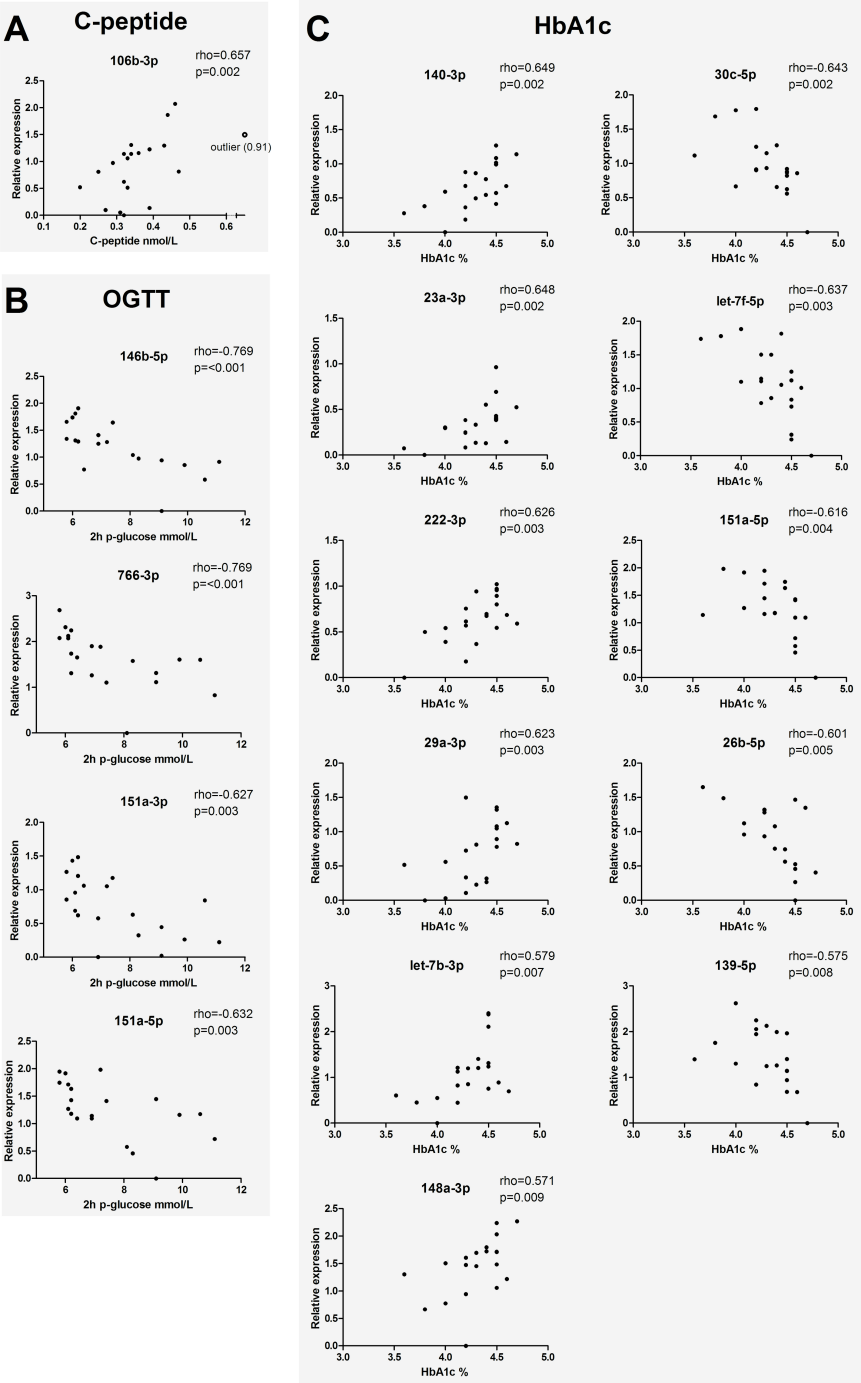
Correlation of miRNA expression to parameters related to glucose homeostasis in the high-risk group. (**A)** fasting C-peptide, **B)** HbA1c and **C)** glucose at 120 min of OGTT. Correlations with p<0.01 have been included in the figure.

**Fig 4:**
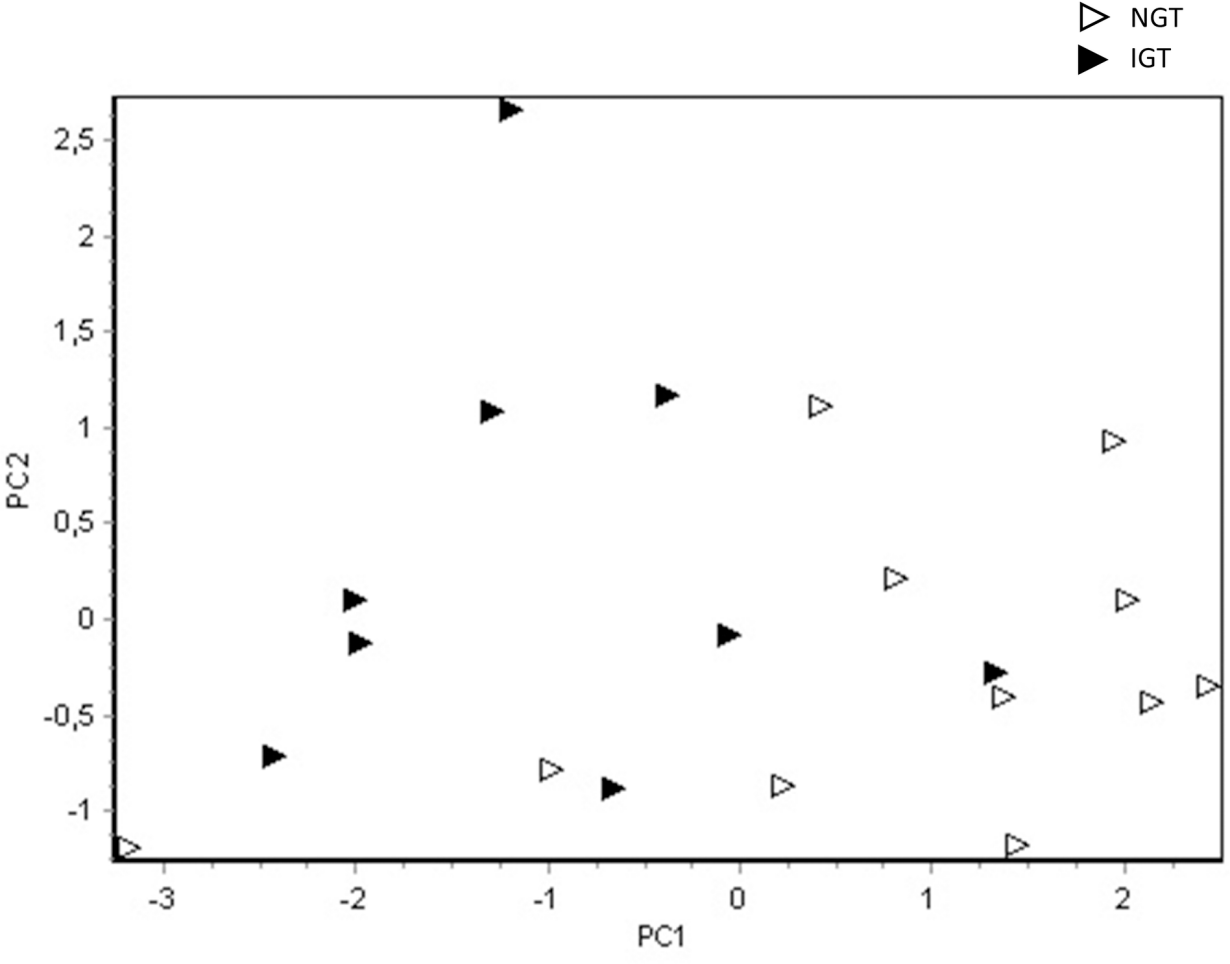
PCA visualization illustrating the separation of high-risk individuals with impaired glucose tolerance from those with normal glucose tolerance. Impaired glucose tolerance: IGT ≥7.8mmol/l at 120min of OGTT (n = 9), normal glucose tolerance: NGT (n = 11). The PCA score plot is based on the 18 miRNAs that correlated to glucose at 120 min of OGTT, with p<0.05.

To assess whether miRNA expression in serum was related to glucose homeostasis within the group of high-risk individuals, correlation analyses were performed between miRNA expression and fasting blood glucose, C-peptide, HbA1c and blood glucose at 120 min of OGTT. This revealed highly significant correlations for several miRNAs. The levels of C-peptide correlated with miR-106b-3p (Fig 3A), while blood glucose concentration measured at 120min of OGTT correlated negatively to miR-146b-5p, miR-766-3p, miR-151a-3p and -5p (Fig 3B). The levels of HbA1c were also associated to several miRNAs, showing positive correlations to miR-140-3p, miR-23a-3p, miR-222-3p, miR-29a-3p, let-7b-3p and miR-148a-3p, and negative associations to miR-30c-5p, let-7f-5p, miR-151a-5p, miR-26b-5p and miR-139-5p were observed (Fig 3C). The miRNAs that correlated to glucose homeostasis with lower statistical significance (0.01<p<0.05) are found in S2 Table. The 18 miRNAs that correlated to glucose levels at 120min of OGTT (p<0.05) were included in a PCA score plot to illustrate the separation of high-risk individuals with (n = 9) and without (n = 11) impaired glucose tolerance (IGT, glucose ≥7.8 mmol/l at 120 min of OGTT, Fig 4).

### miRNAs and islet autoantibodies among high-risk individuals

**Fig 5:**
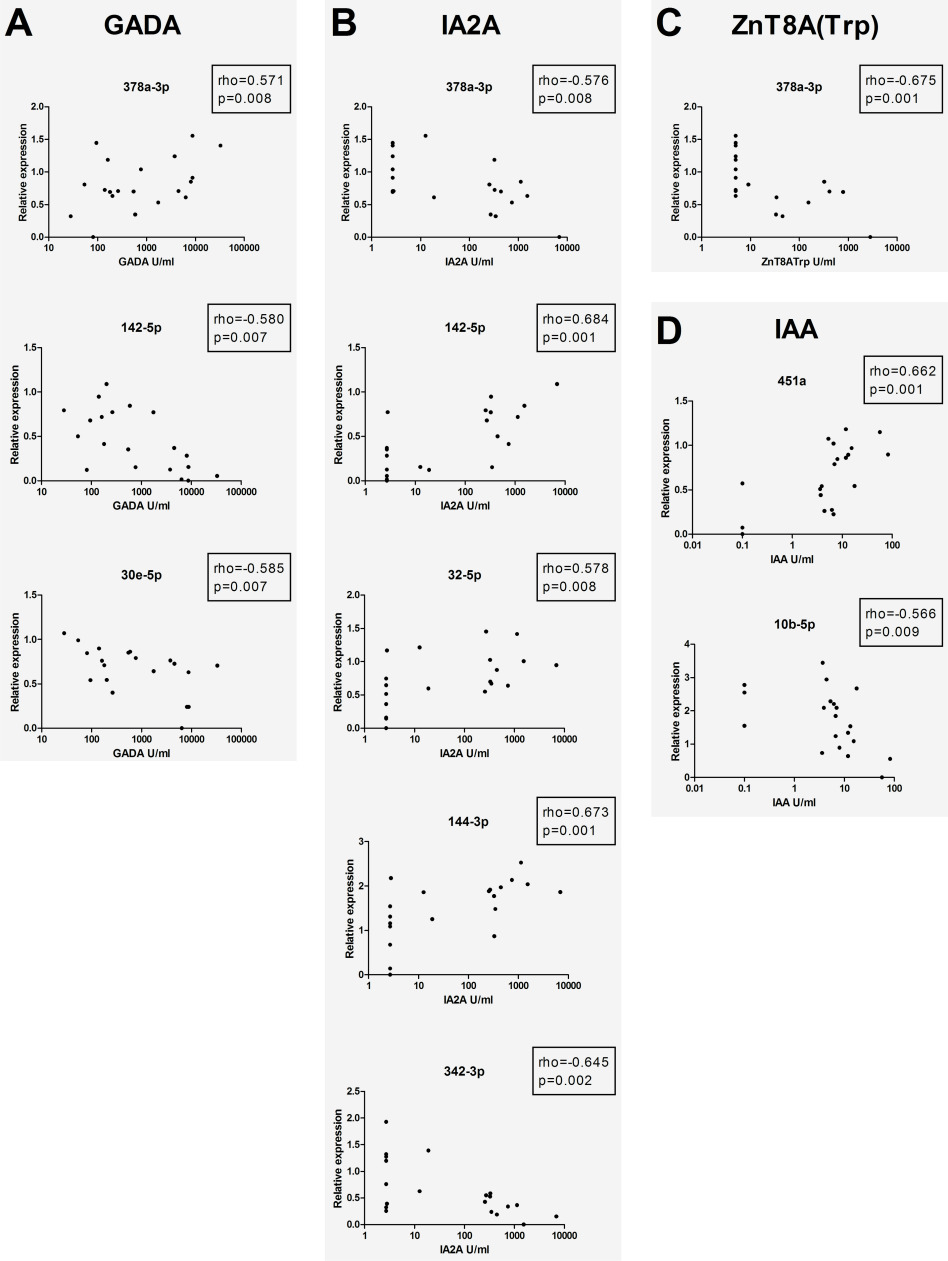
Correlation of miRNA expression to islet autoantibodies within the high-risk group. **A)** GADA, **B)** IA2A, **C)** IAA and **D)** ZnT8A(Trp). X-axes are presented in log10, and only correlations with p<0.01 have been included in the figure.

Analysis of associations between miRNA expression and titers of islet autoantibodies (GADA, IA2A, IAA and the three variants of ZnT8A: Trp/Arg/Glt) revealed highly significant correlations (p<0.01, Fig 5A–5D). GADA titers correlated positively with miR-378a-3p, and negatively with miR-142-5p and miR-30e-3p (Fig 5A), whereas IA2A showed positive correlations to miR-142-5p, miR-144-3p and miR-32-5p, and negative to miR-342-3p and miR-378a-3p (Fig 5B). ZnT8A(Trp) correlated negatively to miR-378a-3p (Fig 5C), while IAA was positively associated to miR-451a and negatively to miR-10b-5p (Fig 5D). A group of miRNAs were also associated to islet autoantibodies with lower statistical significance (0.01<p<0.05, S2 Table).

### Identification of miRNA molecular pathways

Computational screening was performed for the miRNAs associated to glucose homeostasis and autoantibody titers (p<0.01), using miRWalk´s predicted target module. The majority of miRNAs associated to glucose homeostasis had target genes significantly over-represented in pathways related to general cellular processes like endocytosis, but also to more diabetes-relevant pathways like insulin signaling (S3 Table). This was also the case for the majority of miRNAs associated to autoantibody titers (S4 Table).

## Discussion

In this study, serum miRNA profile in autoantibody positive individuals with high risk of T1D did not differ with respect to healthy, age-matched controls. In contrast, many miRNAs differed between high-risk individuals and T1D patients. Considering that the children with increased risk all had ongoing autoimmunity, and in many cases also showed signs of dysglycemia, more similarities to newly diagnosed diabetes patients would have been expected. The high-risk individuals were however asymptomatic at the time of sampling, and from a clinical point of view still healthy. Thus, even though several miRNAs seemed to be related to dysregulated glucose homeostasis, the major metabolic disturbances at the time of T1D diagnosis might explain why the high-risk individuals showed higher resemblance to healthy than to diabetic subjects.

Within the group of risk-children, associations between miRNA expression and different measures of glucose homeostasis were revealed. Among the miRNAs correlating to HbA1c were miR-140-3p, miR-29a-3p and let-7f-5p. They have all previously been associated to different types of diabetes [21], and members of the let-7 family are known to be involved in regulation of glucose metabolism [22]. Of particular interest was the positive association between HbA1c and miR-29a-3p, as it recently was shown in a mouse model for beta cell stress-induced diabetes that this miRNA works as a positive regulator of insulin secretion, with a protective role in disease development [23]. Moreover, upregulation of miR-29a in islets of pre-diabetic NOD mice [15] has been considered as a compensatory mechanism to restore insulin secretion after beta cell loss [23]. Perhaps the association of miR-29a-3p to HbA1c in high-risk individuals in our study is a reflection of such compensatory mechanisms. Glucose levels after OGTT were negatively associated to miR-146b-5p. Downregulation of this miRNA has been described in PBMC from T1D patients [8], but we did not observe any deviation in serum samples from the diabetic group. Other miRNAs associated to OGTT in our study were miR-151a-3p and -5p. It seems likely that miR-151 might be related to metabolic processes, since miR-151a-5p has been connected to mitochondrial activity [24], and serum levels of both 3p and 5p have been reported to decrease after exercise [25]. Given the apparent relation between serum miRNA expression and glucose levels in OGTT, it might be valuable to further explore the feasibility to distinguish high-risk individuals with abnormal glucose tolerance based on miRNA profile, as a complement to the oral glucose tolerance test. The only strong association observed between miRNAs and C-peptide was that with miR-106b-3p, a miRNA known to induce mitochondrial dysfunction and insulin resistance in muscle cells [26], and with putative target genes significantly over-represented in the insulin signaling pathway. To our knowledge, there are no previous reports of possible associations between miR-106b and insulin secretion in either individuals with T1D risk or in T1D patients. Taken together, our results suggest that serum miRNAs may be related to glucose homeostasis during the pre-diabetic period, likely reflecting both causes and consequences of disturbed glucose homeostasis; i.e. disturbed insulin secretion locally in the pancreas and altered glucose metabolism in peripheral tissues like muscle and liver. The pathway interaction analysis also supports that many of the gene targets for miRNAs associated to glucose homeostasis are over-represented in different metabolic pathways.

Another interesting finding was the observed association between several miRNAs and autoantibody titers in high-risk individuals. Of particular interest was the negative correlation of miR-342-3p to IA2A, as it recently has been reported that miR-342-3p directly targets IA2- and IA2β-mRNA [27]. The same study showed that high-glucose stimulation increased the expression of miRNAs associated to autoantigen regulation, and it has been hypothesized that this and other related miRNAs may play a role in maintaining balanced levels of major T1D autoantigens when glucose levels are altered, since the levels of autoantigens like IA2, IA2β and GAD_65_ also are known to increase in response to high glucose [27–29]. A majority of the high-risk individuals in our study showed signs of dysglycemia, regardless of autoantibody profile or whether they later progressed to T1D, thus elevated glucose cannot explain the observed pattern for miR-342-3p. On the other hand, as the progressors had higher IA2A titers [18], perhaps a failure to increase miR-342-3p has resulted in higher autoantigen concentrations which may accelerate the autoimmune process, ultimately leading to clinical disease. Additional evidence for a role of miR-342 in T1D has been provided by results showing decreased levels in regulatory T cells from diabetic patients [12], and downregulation of miR-342-3p in PBMC from type 1- compared to type 2 diabetes patients [21]. It is possible that underlying connections between glucose homeostasis and the level of ongoing islet autoimmunity may explain some of the observed miRNA-autoantibody associations. For instance, miR-144, positively associated to IA2A in our study, has been demonstrated to impair insulin signaling and to be linearly upregulated with increasing glycemic status [30]. Also miR-378, which was negatively correlated to IA2A- and ZnT8A(Trp) but positively to GADA, has been ascribed a role in metabolic processes [31], e.g. as a regulator of mitochondrial metabolism and systemic energy homeostasis [32]. The shared association of miR-378a-3p with IA2A and ZnT8A may be related to the fact that IA2A- and ZnT8A titers were positively associated in the same samples [18], which is in line with previous reports on high overlap between IA2A and ZnT8A positivity in T1D [33]. Furthermore, an inverse association between GADA and IA2A in the same individuals might explain the opposite expression seen for miR-378a-3p and miR-142-5p in relation to GADA- and IA2A titers. To our knowledge, there are no previous studies reporting associations between islet autoantibodies and miRNA expression in serum from high-risk individuals or T1D patients, although such connections have been investigated in PBMC from T1D patients [8, 11].

The miRNA profiles of T1D subjects deviated substantially both from healthy controls and high-risk individuals. Several of these miRNAs are biologically relevant in beta cell physiology/pathology [34, 35] and many of them have previously been described as dysregulated in T1D patients, e.g. upregulation of miR-34a, miR-342-3p [36], miR-152, miR-181a and miR-148a [13] and downregulation of miR-107 [36]. There are however some discrepancies between the observed results, since for instance miR-24 and miR-25 were upregulated in T1D in a previous study [13] while we found lower expression in T1D patients. This might be explained by differences in age and sampling time between the studied T1D cohorts [13, 36]. Beside these differences we have to be cautious when interpreting our data because of other limitations, as the T1D subjects were included as a reference group for the high-risk individuals and consisted of only eight samples, and since effects of the different storage temperatures cannot be excluded. However, the remarkable stability of miRNA [6], together with the fact that our results to a great extent are in agreement with findings from previous studies on T1D patients [13, 36], support that storage did not have a major impact on the miRNA quality.

We have previously shown that the children who progressed to manifest diabetes (progressors) had higher levels of IA2A and ZnT8A than non-progressors, but there was no statistical difference in the number of different autoantibodies to which progressors and non-progressors were positive to [18]. In addition, there was no difference in the prevalence of HLA-genotypes associated to increased risk between progressors and non-progressors. As there were no differences with regard to neither number of autoantibodies nor HLA-type we do not expect miRNA levels to be influenced by these factors. When investigating individuals with high HLA risk and low HLA risk within the high-risk group, a few miRNAs differed in relation to HLA risk, but due to the low number of individuals it was difficult to draw conclusions. The high-risk group may have been too small to be able to detect subtle differences in miRNA expression in relation to healthy controls and T1D patients. However, large cohorts of autoantibody-positive individuals identified from the general population are rare, because of the screening complexity. Despite the limited size, the group was very homogenous with regard to age, and the healthy controls were well matched to the high-risk group in terms of both age and gender. The limited group size may also explain why we found only a few slightly deviating miRNAs and not a specific miRNA profile that was related to disease progression when comparing high-risk individuals progressing or not to T1D. It would be interesting to investigate in other risk-cohorts whether these particular miRNAs may be relevant for prediction of disease progression.

## Conclusion

No specific miRNA profile was identified for individuals at high risk of T1D, nor could risk-individuals progressing to clinical disease be clearly distinguished from non-progressors based on serum miRNA expression. However, several miRNAs appeared to be tightly associated to glucose homeostasis and islet autoantibodies among high-risk individuals, and these possible associations may be useful in the stratification of risk. Larger studies in other high-risk cohorts are warranted to corroborate these findings and to further assess the possibilities of using circulating miRNAs in T1D risk stratification.

## Supporting information

S1 Table miRNAs included in exiqons 384-well miRCURY LNA^TM^ Universal RT microRNA PCR Serum/Plasma Focus Panels, V3. (PDF)

S2 Table Correlations (0.01<p<0.05) between miRNAs and biochemical parameters, among T1D high-risk individuals. (PDF)

S3 Table Predicted targets for miRNAs correlated to measures of glucose homeostasis were significantly over-represented in several KEGG pathways and GO biological processes. Functionally closely related KEGG- and GO-terms have been clustered, and only pathways/processes showing significant over-representation in both databases have been included. X represents significant over-representation for the particular pathway/process, and the sign before the clinical measure implies the direction of the correlation to the miRNA.
(PDF)

S4 Table Predicted targets for miRNAs correlated to autoantibody titers were significantly over-represented in several KEGG pathways and GO biological processes. Functionally closely related KEGG- and GO-terms have been clustered, and only pathways/processes showing significant over-representation in both databases have been included. X represents significant over-representation for the particular pathway/process.
(PDF)

S1 File Median miRNA levels for 129 miRNAs and p values (Mann-Whitney U-test) from comparisons of high-risk individuals (n = 20) and healthy controls (n = 17), high-risk individuals and patients with T1D (n = 8), healthy controls and T1D patients, as well as progressors (n = 11) and non-progressors (n = 9). (XLSX)

S2 File Data set. (XLSX)
