# Supplementary material for: Serum miRNA levels are related to glucose homeostasis and islet autoantibodies in children with high risk for type 1 diabetes
Source: PLoS One. 2018 Jan 18;13(1):e0191067. doi: 10.1371/journal.pone.0191067 (PMC5773164; doi:10.1371/journal.pone.0191067)
Supplement: S1 Table — (PDF) [file pone.0191067.s001.pdf]

miRname (human)	microRNA target sequence	miRname (human)	microRNA target sequence
hsa-let-7a-5p	UGAGGUAGUAGGUUGUAUAGUU	hsa-miR-185-5p	UGGAGAGAAAGGCAGUUCCUGA
hsa-let-7b-3p	CUAUACAACCUACUGCCUUCCC	hsa-miR-186-5p	CAAAGAAUUCUCCUUUUGGGCU
hsa-let-7b-5p	UGAGGUAGUAGGUUGUGUGGUU	hsa-miR-18a-3p	ACUGCCCUAAGUGCUCCUUCUGG
hsa-let-7c	UGAGGUAGUAGGUUGUAUGGUU	hsa-miR-18a-5p	UAAGGUGCAUCUAGUGCAGAUAG
hsa-let-7d-3p	CUAUACGACCUGCUGCCUUUCU	hsa-miR-18b-5p	UAAGGUGCAUCUAGUGCAGUUAG
hsa-let-7d-5p	AGAGGUAGUAGGUUGCAUAGUU	hsa-miR-190a	UGAUAUGUUUGAUAUAUUAGGU
hsa-let-7e-5p	UGAGGUAGGAGGUUGUAUAGUU	hsa-miR-191-5p	CAACGGAAUCCCAAAAGCAGCUG
hsa-let-7f-5p	UGAGGUAGUAGAUUGUAUAGUU	hsa-miR-192-5p	CUGACCUAUGAAUUGACAGCC
hsa-let-7g-5p	UGAGGUAGUAGUUUGUACAGUU	hsa-miR-193b-3p	AACUGGCCCUCAAAGUCCCGCU
hsa-let-7i-3p	CUGCGCAAGCUACUGCCUUGCU	hsa-miR-194-5p	UGUAACAGCAACUCCAUGUGGA
hsa-let-7i-5p	UGAGGUAGUAGUUUGUGCUGUU	hsa-miR-195-5p	UAGCAGCACAGAAAUAUUGGC
hsa-miR-1	UGGAAUGUAAAGAAGUAUGUAU	hsa-miR-197-3p	UUCACCACCUUCUCCACCCAGC
hsa-miR-101-3p	UACAGUACUGUGAUAACUGAA	hsa-miR-199a-3p	ACAGUAGUCUGCACAUUGGUUA
hsa-miR-103a-3p	AGCAGCAUUGUACAGGGCUAUGA	hsa-miR-199a-5p	CCCAGUGUUCAGACUACCUGUUC
hsa-miR-106a-5p	AAAAGUGCUUACAGUGCAGGUAG	hsa-miR-19a-3p	UGUGCAAAUCUAUGCAAAACUGA
hsa-miR-106b-3p	CCGCACUGUGGGUACUUGCUGC	hsa-miR-19b-3p	UGUGCAAAUCCAUGCAAAACUGA
hsa-miR-106b-5p	UAAAGUGCUGACAGUGCAGAU	hsa-miR-200a-3p	UAACACUGUCUGGUAACGAUGU
hsa-miR-107	AGCAGCAUUGUACAGGGCUAUGA	hsa-miR-200c-3p	UAAUACUGCCGGGUAAUGAUGGA
hsa-miR-10a-5p	UACCCUGUAGAUCCGAAUUUGUG	hsa-miR-204-5p	UUCCCUUUGUCAUCCUAUGCCU
hsa-miR-10b-5p	UACCCUGUAGAACCGAAUUUGUG	hsa-miR-205-5p	UCCUUCAUUCCACCGGAGUCUG
hsa-miR-122-5p	UGGAGUGUGACAAUGGUGUUUG	hsa-miR-208a	AUAAGACGAGCAAAAAGCUUGU
hsa-miR-125a-5p	UCCCUGAGACCCUUUAACCUGUGA	hsa-miR-20a-3p	ACUGCAUUAUGAGCACUUAAAG
hsa-miR-125b-5p	UCCCUGAGACCCUAACUUGUGA	hsa-miR-20a-5p	UAAAGUGCUUAUAGUGCAGGUAG
hsa-miR-126-3p	UCGUACCGUGAGUAAUAAUGCG	hsa-miR-20b-5p	CAAAGUGCUCAUAGUGCAGGUAG
hsa-miR-127-3p	UCGGAUCCGUCUGAGCUUGGCU	hsa-miR-210	CUGUGCGUGUGACAGCGGCUGA
hsa-miR-128	UCACAGUGAACCGGUCUCUUU	hsa-miR-2110	UUGGGGAAACGGCCGCUGAGUG
hsa-miR-130a-3p	CAGUGCAAUGUUAAAAGGGCAU	hsa-miR-215	AUGACCUAUGAAUUGACAGAC
hsa-miR-130b-3p	CAGUGCAAUGAUGAAAGGGCAU	hsa-miR-21-5p	UAGCUUAUCAGACUGAUGUUGA
hsa-miR-132-3p	UAACAGUCUACAGCCAUGGUCG	hsa-miR-221-3p	AGCUACAUUGUCUGCUGGGUUUC
hsa-miR-133a	UUUGGUCCCCUUCAACCAGCUG	hsa-miR-222-3p	AGCUACAUCUGGCUACUGGGU
hsa-miR-133b	UUUGGUCCCCUUCAACCAGCUG	hsa-miR-223-3p	UGUCAGUUUGUCAAAUACCCCA
hsa-miR-136-5p	ACUCCAUUUGUUUUGAUGAUGGA	hsa-miR-223-5p	CGUGUAUUUGACAAGCUGAGUU
hsa-miR-139-5p	UCUACAGUGCACGUGUCUCCAGU	hsa-miR-22-3p	AAGCUGCCAGUUGAAGAACUGU
hsa-miR-140-3p	UACCACAGGGUAGAACCACGG	hsa-miR-22-5p	AGUUCUUCAGUGGCAAGCUUUA
hsa-miR-140-5p	CAGUGGUUUUACCCUAUGGUAG	hsa-miR-23a-3p	AUCACAUUGCCAGGGAUUUCC
hsa-miR-141-3p	UAACACUGUCUGGUAAAGAUGG	hsa-miR-23b-3p	AUCACAUUGCCAGGGAUUACC
hsa-miR-142-3p	UGUAGUGUUUCCUACUUUAUGGA	hsa-miR-24-3p	UGGCUCAGUUCAGCAGGAACAG
hsa-miR-142-5p	CAUAAAGUAGAAAGCACUACU	hsa-miR-25-3p	CAUUGCACUUGUCUCGGUCUGA
hsa-miR-143-3p	UGAGAUGAAGCACUGUAGCUC	hsa-miR-26a-5p	UUCAAGUAAUCCAGGAUAGGCU
hsa-miR-144-3p	UACAGUAUAGAUGAUGUACU	hsa-miR-26b-5p	UUCAAGUAAUUCAGGAUAGGU
hsa-miR-144-5p	GGAUAUCAUCAUAUACUGUAAG	hsa-miR-27a-3p	UUCACAGUGGCUAAGUUCCGC
hsa-miR-145-5p	GUCCAGUUUUCCCAGGAAUCCCU	hsa-miR-27b-3p	UUCACAGUGGCUAAGUUCUGC
hsa-miR-146a-5p	UGAGAACUGAAUUCCAUGGGUU	hsa-miR-28-3p	CACUAGAUUGUGAGCUCCUGGA
hsa-miR-146b-5p	UGAGAACUGAAUUCCAUAGGCU	hsa-miR-28-5p	AAGGAGCUCACAGUCUAUUGAG
hsa-miR-148a-3p	UCAGUGCACUACAGAACUUUGU	hsa-miR-296-5p	AGGGCCCCCCCUCAAUCCUGU
hsa-miR-148b-3p	UCAGUGCAUCACAGAACUUUGU	hsa-miR-29a-3p	UAGCACCAUCUGAAAUCGGUUA
hsa-miR-150-5p	UCUCCCAACCCUUGUACCAGUG	hsa-miR-29a-5p	ACUGAUUUCUUUUGGUGUUCAG
hsa-miR-151a-3p	CUAGACUGAAGCUCCUUGAGG	hsa-miR-29b-2-5p	CUGGUUUCACAUGGUGGCUUAG
hsa-miR-151a-5p	UCGAGGAGCUCACAGUCUAGU	hsa-miR-29b-3p	UAGCACCAUUUGAAAUCAGUGUU
hsa-miR-152	UCAGUGCAUGACAGAACUUGG	hsa-miR-29c-3p	UAGCACCAUUUGAAAUCGGUUA
hsa-miR-154-5p	UAGGUUAUCCGUGUUGCCUUCG	hsa-miR-301a-3p	CAGUGCAAUAGUAUUGUCAAAGC
hsa-miR-155-5p	UUAAUGCUAAUCGUGAUAGGGGU	hsa-miR-30a-5p	UGUAAACAUCCUCGACUGGAAG
hsa-miR-15a-5p	UAGCAGCACAUAAUGGUUUGUG	hsa-miR-30b-5p	UGUAAACAUCCUACACUCAGCU
hsa-miR-15b-3p	CGAAUCAUUAUUUGCUGCUCUA	hsa-miR-30c-5p	UGUAAACAUCCUACACUCUCAGC
hsa-miR-15b-5p	UAGCAGCACAUCAUGGUUUACA	hsa-miR-30d-5p	UGUAAACAUCCCCGACUGGAAG
hsa-miR-16-2-3p	CCAAUAUUACUGUGCUGCUUUA	hsa-miR-30e-3p	CUUUCAGUCGGAUGUUUACAGC
hsa-miR-16-5p	UAGCAGCACGUAAAUAUUGGCG	hsa-miR-30e-5p	UGUAAACAUCCUUGACUGGAAG
hsa-miR-17-5p	CAAAGUGCUUACAGUGCAGGUAG	hsa-miR-320a	AAAAGCUGGGUUGAGAGGGCGA
hsa-miR-181a-5p	AACAUUCAACGCUGUCGGUGAGU	hsa-miR-320b	AAAAGCUGGGUUGAGAGGGCAA
hsa-miR-182-5p	UUUGGCAAUGGUAGAACUCACACU	hsa-miR-324-3p	ACUGCCCCAGGUGCUGCUGG

miRname (human)	microRNA target sequence
hsa-miR-324-5p	CGCAUCCCCUAGGGCAUUGGUGU
hsa-miR-32-5p	UAUUGCACAUUACUAAGUUGCA
hsa-miR-326	CCUCUGGGCCCUUCCUCCAG
hsa-miR-328	CUGGCCCUCUCUGCCCUUCCGU
hsa-miR-331-3p	GCCCCUGGGCCUAUCCUAGAA
hsa-miR-335-5p	UCAAGAGCAAUAACGAAAAAUGU
hsa-miR-338-3p	UCCAGCAUCAGUGAUUUUGUUG
hsa-miR-339-3p	UGAGCGCCUCGACGACAGAGCCG
hsa-miR-339-5p	UCCCUGUCCUCCAGGAGCUCACG
hsa-miR-33a-5p	GUGCAUUGUAGUUGCAUUGCA
hsa-miR-342-3p	UCUCACACAGAAAUCGCACCCGU
hsa-miR-346	UGUCUGCCCGCAUGCCUGCCUCU
hsa-miR-34a-5p	UGGCAGUGUCUUAGCUGGUUGU
hsa-miR-361-3p	UCCCCCAGGUGUGAUUCUGAUUU
hsa-miR-363-3p	AAUUGCACGGUAUCCAUCUGUA
hsa-miR-365a-3p	UAAUGCCCCUAAAAAUCCUUAU
hsa-miR-374a-5p	UUAUAAUACAACCUGAUAAGUG
hsa-miR-374b-5p	AUAUAAUACAACCUGCUAAGUG
hsa-miR-375	UUUGUUCGUUCGGCUCGCGUGA
hsa-miR-376a-3p	AUCAUAGAGGAAAAUCCACGU
hsa-miR-378a-3p	ACUGGACUUGGAGUCAGAAGG
hsa-miR-382-5p	GAAGUUGUUCGUGGUGGAUUCG
hsa-miR-409-3p	GAAUGUUGCUCGGUGAACCCCU
hsa-miR-421	AUCAACAGACAUUAAUUGGGCGC
hsa-miR-423-3p	AGCUCGGUCUGAGGCCCCUCAGU
hsa-miR-423-5p	UGAGGGGCAGAGAGCGAGACUUU
hsa-miR-424-5p	CAGCAGCAAUUCAUGUUUUGAA
hsa-miR-425-3p	AUCGGGAAUGUCGUGUCCGCCC
hsa-miR-425-5p	AAUGACACGAUCACUCCCGUUGA
hsa-miR-451a	AAACCGUUACCAUUACUGAGUU
hsa-miR-484	UCAGGCUCAGUCCCCUCCCGAU
hsa-miR-485-3p	GUCAUACACGGCUCUCCUCUCU
hsa-miR-486-5p	UCCUGUACUGAGCUGCCCCGAG
hsa-miR-495-3p	AAACAAACAUGGUGCACUUCUU
hsa-miR-497-5p	CAGCAGCACACUGUGGUUUGU
hsa-miR-500a-5p	UAAUCCUUGCUACCUGGGUGAGA
hsa-miR-501-3p	AAUGCACCCGGGCAAGGAUUCU
hsa-miR-502-3p	AAUGCACCUGGGCAAGGAUUCA
hsa-miR-505-3p	CGUCAACACUUGCUGGUUUCCU
hsa-miR-532-3p	CCUCCCACACCCAAGGCUUGCA
hsa-miR-532-5p	CAUGCCUUGAGUGUAGGACCGU
hsa-miR-543	AAACAUUCGCGGUGCACUUCUU
hsa-miR-551b-3p	GCGACCCAUACUUGGUUUCAG
hsa-miR-574-3p	CACGCUCAUGCACACACCCACA
hsa-miR-584-5p	UUAUGGUUUGCCUGGGACUGAG
hsa-miR-590-5p	GAGCUUAUUCAUAAAAGUGCAG
hsa-miR-605	UAAAUCCCAUGGUGCCUUCUCCU
hsa-miR-629-5p	UGGGUUUACGUUGGGAGAACU
hsa-miR-652-3p	AAUGGCGCCACUAGGGUUGUG
hsa-miR-660-5p	UACCCAUUGCAUAUCGGAGUUG
hsa-miR-766-3p	ACUCCAGCCCCACAGCCUCAGC
hsa-miR-885-5p	UCCAUUACACUACCCUGCCUCU
hsa-miR-92a-3p	UAUUGCACUUGUCCCGGCCUGU
hsa-miR-92b-3p	UAUUGCACUCGUCCCGGCCUCC
hsa-miR-93-3p	ACUGCUGAGCUAGCACUUCCCG
hsa-miR-93-5p	CAAAGUGCUGUUCGUGCAGGUAG
hsa-miR-95	UUCAACGGGUAUUUAUUGAGCA
hsa-miR-99a-5p	AACCCGUAGAUCCGAUCUUGUG
hsa-miR-99b-5p	CACCCGUAGAACCGACCUUGCG
