# Supplementary material for: Serum miRNA levels are related to glucose homeostasis and islet autoantibodies in children with high risk for type 1 diabetes
Source: PLoS One. 2018 Jan 18;13(1):e0191067. doi: 10.1371/journal.pone.0191067 (PMC5773164; doi:10.1371/journal.pone.0191067)
Supplement: S2 Table — (PDF) [file pone.0191067.s002.pdf]

miRNAs correlated to glucose homeostasis (p<0.05, >0.01, Spearman correlation)

C-peptide			HbA1c			Fasting blood glucose			Blood glucose, 120 min OGTT		
miRNA	*rho*	*p*	*miRNA*	*rho*	*p*	*miRNA*	*rho*	*p*	*miRNA*	*rho*	*p*
29b-3p	-0.489	0.029	103a-3p	-0.555	0.011	194-5p	0.469	0.037	221-3p	-0.552	0.012
223-5p	0.482	0.032	28-5p	-0.535	0.015				19a-3p	0.535	0.015
32-5p	-0.467	0.038	let-7a-5p	-0.529	0.016				326	-0.519	0.019
			30b-5p	-0.525	0.018				146a-5p	-0.517	0.020
			423-5p	0.522	0.018				29c-3p	0.505	0.023
			17-5p	-0.516	0.020				424-5p	0.491	0.028
			365a-3p	0.509	0.022				101-3p	0.488	0.029
			378a-3p	0.509	0.022				505-3p	0.483	0.031
			502-3p	0.485	0.030				423-3p	-0.481	0.032
			150-5p	0.483	0.031				154-5p	0.474	0.035
			18b-5p	-0.475	0.034				15a-5p	0.467	0.038
			424-5p	0.460	0.041				19b-3p	0.467	0.038
			320a	0.459	0.042				378a-3p	0.462	0.040
			29c-3p	0.455	0.044				103a-3p	-0.461	0.041

miRNAs correlated to islet autoantibodies (p<0.05, >0.01, Spearman correlation)

GADA			IA-2A			IAA			ZnT8Trp			ZnT8Glut		
miRNA	*rho*	*p*	*miRNA*	*rho*	*p*	*miRNA*	*rho*	*p*	*miRNA*	*rho*	*p*	*miRNA*	*rho*	*p*
148b-3p	0.511	0.021	320b	-0.561	0.010	194-5p	0.566	0.011	424-5p	-0.558	0.011	424-5p	-0.531	0.016
144-3p	-0.502	0.024	421	-0.564	0.010	16-5p	0.540	0.014	29c-3p	-0.535	0.015	378a-3p	-0.491	0.028
106b-5p	-0.468	0.038	423-5p	-0.538	0.014	99b-5p	-0.515	0.020	423-5p	-0.537	0.015	23a-3p	-0.487	0.029
let-7b-5p	-0.453	0.045	92a-3p	-0.529	0.017	30d-5p	-0.454	0.044	374a-5p	0.528	0.017	423-5p	-0.475	0.034
			let-7g-5p	0.505	0.023				505-3p	-0.509	0.022			
			374a-5p	0.492	0.028				425-5p	-0.505	0.023			
			142-3p	0.489	0.029				101-3p	-0.495	0.026			
			222-3p	-0.475	0.034				92a-3p	-0.489	0.029			
			let-7b-3p	-0.467	0.038				374b-5p	0.486	0.030			
			148b-3p	-0.453	0.045				16-2-3p	-0.477	0.033			
									197-3p	-0.477	0.033			
									let-7b-3p	-0.459	0.042			
