# Supplementary material for: Serum miRNA levels are related to glucose homeostasis and islet autoantibodies in children with high risk for type 1 diabetes
Source: PLoS One. 2018 Jan 18;13(1):e0191067. doi: 10.1371/journal.pone.0191067 (PMC5773164; doi:10.1371/journal.pone.0191067)
Supplement: S3 Table — Functionally closely related KEGG- and GO-terms have been clustered, and only pathways/processes showing significant over-representation in both databases have been included. X represents significant over-representation for the particular pathway/process, and the sign before the clinical measure implies the direction of the correlation to the miRNA. (PDF) [file pone.0191067.s003.pdf]

Direction of the correlation to each miRNA (+/-)

**KEGG pathways or GO biological processes related to:**	**miR-106b-3p**	**miR-146b-5p**	**miR-766-3p**	**miR-151a-3p**	**miR-151a-5p**	**miR-30c-5p**	**let-7f-5p**	**miR-26b-5p**	**miR-139-5p**	**miR-140-3p**	**miR-23a-3p**	**miR-222-3p**	**miR-29a-3p**	**let-7b-3p**	**miR-148a-3p**
C-peptide	+														
OGTT		-	-	-	-										
HbA1c					-	-	-	-	-	-	+	+	+	+	+
Axon guidance, axon extension	x	x	x	x	x	x	x	x	x	x	x	x	x	x	x
Endocytosis	x	x	x	x	x	x	x	x	x	x	x	x	x	x	x
Insulin signaling pathway, insulin receptor signaling pathway	x	x	x	x		x		x	x	x	x	x	x	x	x
Calcium signaling, calcium ion transport, calcium ion dependent exocytosis	x	x	x		x	x	x	x	x			x	x	x	x
Apoptosis, apoptotic process	x	x		x	x		x	x	x		x	x	x	x	
MAPK signaling pathway, MAPK cascade	x	x		x	x	x	x		x	x	x			x	
Phosphatidylinositol signaling system, phosphatidylinositol phosphorylation			x	x	x	x	x		x	x		x	x		x
Cell adhesion molecules CAMs, cell adhesion	x		x		x	x	x		x	x	x			x	
T cell receptor signaling pathway		x			x		x	x		x		x	x		
Wnt signaling pathway, wnt receptor signaling pathway		x	x				x				x		x	x	x
Regulation of actin cytoskeleton, regulation of actin cytoskeleton organization				x		x				x			x	x	x
Adherens junction, adherens junction assembly, adherens junction organization	x	x	x						x						x
B cell receptor signaling pathway, B cell activation, B cell differentiation				x	x		x		x	x					
Leucocyte transendotelial migration, leukocyte migration	x		x						x		x	x			
Cell cycle							x	x						x	x
Tight junction, tight junction assembly	x	x									x	x			
Toll like receptor signaling pathway	x	x					x						x		
Adipocytokine signaling pathway, adipose tissue development			x	x									x		
Glycerophospholipid metabolism, glycerophospholipid biosynthetic process			x				x								x
Lysosome, lysosome organization, lysosomal transport					x				x	x					
Chondroitin sulfate metabolic process, chondroitin sulfate biosynthesis													x		x
JAK STAT signaling pathway, JAK STAT cascade				x			x								
Notch signaling pathway												x		x	
Focal adhesion, focal adhesion assembly						x									
Gap junction			x												
Keratan sulfate biosynthesis								x							
Neurotrophin signaling pathway, neutrophil mediated immunity							x								
Peroxisome, peroxisome organization							x								
Pyruvate metabolism, pyruvate metabolic process												x			
