# Supplementary material for: Serum miRNA levels are related to glucose homeostasis and islet autoantibodies in children with high risk for type 1 diabetes
Source: PLoS One. 2018 Jan 18;13(1):e0191067. doi: 10.1371/journal.pone.0191067 (PMC5773164; doi:10.1371/journal.pone.0191067)
Supplement: S4 Table — Functionally closely related KEGG- and GO-terms have been clustered, and only pathways/processes showing significant over-representation in both databases have been included. X represents significant over-representation for the particular pathway/process. (PDF) [file pone.0191067.s004.pdf]

Direction of the correlation to each miRNA (+/-)

	miR-378a-3p	miR-142-5p	miR-30e-5p	miR-342-3p	miR-32-5p	miR-144-3p	miR-451a	miR-10b-5p
GADA	+	-	-					
IA2A	-	+		-	+	+		
IAA							+	-
ZnT8A	-							

KEGG pathways or GO biological processes related to:

	miR-378a-3p	miR-142-5p	miR-30e-5p	miR-342-3p	miR-32-5p	miR-144-3p	miR-451a	miR-10b-5p
Axon guidance	x	x	x	x	x	x	x	x
Endocytosis	x	x	x	x	x	x	x	x
Insulin signaling pathway, insulin receptor signaling pathway, insulin secretion	x	x	x	x	x	x	x	x
Calcium signaling, calcium ion transport	x	x	x		x		x	x
Cell adhesion molecules CAMs, cell adhesion	x	x		x	x	x		x
Regulation of actin cytoskeleton, regulation of actin cytoskeleton organization		x	x	x	x	x		
Apoptosis, apoptotic process	x	x	x	x				
MAPK signaling pathway, MAPK cascade			x	x		x	x	
B cell receptor signaling pathway	x	x		x				
Leukocyte transendothelial migration, leukocyte migration	x		x			x		
Phosphatidylinositol signaling, phosphatidylinositol biosynthetic process, phosphatidylinositol phosphorylation			x		x	x		
Adherens junction, adherens junction organization		x				x		
Adipocytokine signaling pathway, adipose tissue development		x		x				
Fatty acid metabolism, fat cell differentiation		x		x				
Inositol phosphate metabolism, inositol phosphate dephosphorylation, inositol phosphate mediated signaling		x	x					
JAK STAT signaling pathway, JAK STAT cascade	x							x
Notch signaling pathway	x			x				
Cell cycle						x		
Chondroitin sulfate biosynthesis, chondroitin sulfate metabolic process								x
Glycerophospholipid metabolism, glycerophospholipid biosynthetic process	x							
